# Parts Per Trillion Detection of 7-Aminonitrazepam by Nano-Enhanced ELISA

**DOI:** 10.3390/ijms141019474

**Published:** 2013-09-25

**Authors:** Chifang Peng, Xiaohui Duan, Shanshan Song, Feng Xue

**Affiliations:** 1 State Key Lab of Food Science and Technology, School of Food Science and Technology, Jiangnan University, Wuxi 214122, China; E-Mails: 6120111005@vip.jiangnan.edu.cn (X.D.); sss@jiangnan.edu.cn (S.S.); 2 Animal, Plant & Food Inspection Center, Jiangsu Entry-Exit Inspection and Quarantine Bureau, Nanjing 200001, China; E-Mail: fengxue1219@aliyun.com

**Keywords:** 7-aminonitrazepam, ELISA, gold nanoparticle, bioconjugate

## Abstract

It is challenging to detect 7-aminonitrazepam (7-ANZP) residue in animal tissues simply and sensitively by the enzyme-linked sorbent immunoassay (ELISA) method. This paper demonstrates that utilizing a bioconjugate of gold nanoparticles and enzyme-labeled antibody as a signal probe increases the sensitivity of a traditional ELISA for 7-ANZP by nearly 20 times. The sensitivity of this ELISA for 7-ANZP was 5.6 pg/mL in buffer, and the limit of detection (LOD) of 0.18 μg/kg for 7-ANZP in urine could be achieved after the urine samples were simply hydrolyzed and diluted by buffer. This simple and sensitive method has potential application for improving the sensitivity of ELISA methods against various small molecules.

## 1. Introduction

Benzodiazepines are among the most widely prescribed psychotropic drugs, and can eliminate erethism, manic anxiety and have a calming effect on patients by selectively inhibiting unrest and tension. In general, benzodiazepines act as hypnotics in high doses, anxiolytics in moderate doses, and sedatives in low doses [1]. In China, where many young people suffer from sleeplessness with the ever-increasing pressure of work, benzodiazepines are mainly used in the treatment of clinical insomnia [2]. Nitrazepam is one of benzodiazepine derivatives and 7-aminonitrazepam (7-ANZP) is the primary urinary metabolite of nitrazepam [3]. Developing sensitive detection of 7-ANZP will be very useful in clinical analysis, pharmacology, toxicology and forensic science, *etc.* Although there are many methods such as GC/MS and LC/MS/MS [4–8] used in simultaneously detecting varied benzodiazepines (including 7-ANZP), these methods are very laborious, time-consuming and costly. Immunoassays usually possess many advantages compared with these chromatography methods, such as simple, rapid time to result and low cost. ELISA methods for clonazepam (one of benzodiazepines), its main metabolite, 7-aminoclonazepam, and many other benzodiazepines have already been developed [1,4,9], but the limit of detection (LOD) in these methods all were around ppb level. Based on fluorescent CdTe nanoparticle bioconjugations and microfluidic chip, we previously reported an immunoassay for the metabolite of clonazepam. In this method, the LOD achieved was 0.2 ng/mL [10].

Although the above-mentioned immunoassays have shown very good results, detecting benzodiazepines in urine using simple and sensitive ELISA methods is challenging due to serious matrix interference [11–14]. Fortunately, the matrix interference in many ELISA methods can be avoided by diluting the sample sufficiently [11,13,15]. This strategy may be the only way to develop highly sensitive ELISA methods

**Scheme 1:**
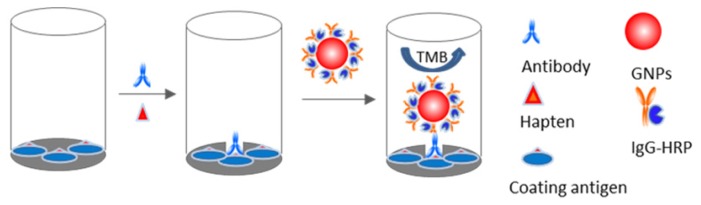
Nano-enhanced ELISA method.

Gold nanoparticles (GNPs) with outstanding characteristics have attracted great interest for applications in biosensors in recent years [11,16–18]. GNPs can conveniently be coated with functional biological molecules (antibody, oligonucleotide and enzyme) for use in highly sensitive biosensors. In this work, a nano-enhanced ELISA was demonstrated for rapid and sensitive detection of 7-ANZP in urine samples. Scheme 1 shows the principle of this nano-enhanced ELISA method. Combining a traditional ELISA format and a GNP-antibody-enzyme bioconjugate as a single probe, this nano-enhanced ELISA method achieved a remarkably low detection limit of 0.18 ng/g 7-ANZP in urine using a simple traditional ELISA protocol.

## 2. Results and Discussion

### 2.1. Preparation of 7-Aminonitrazepam -Protein Conjugate

**Figure 1:**
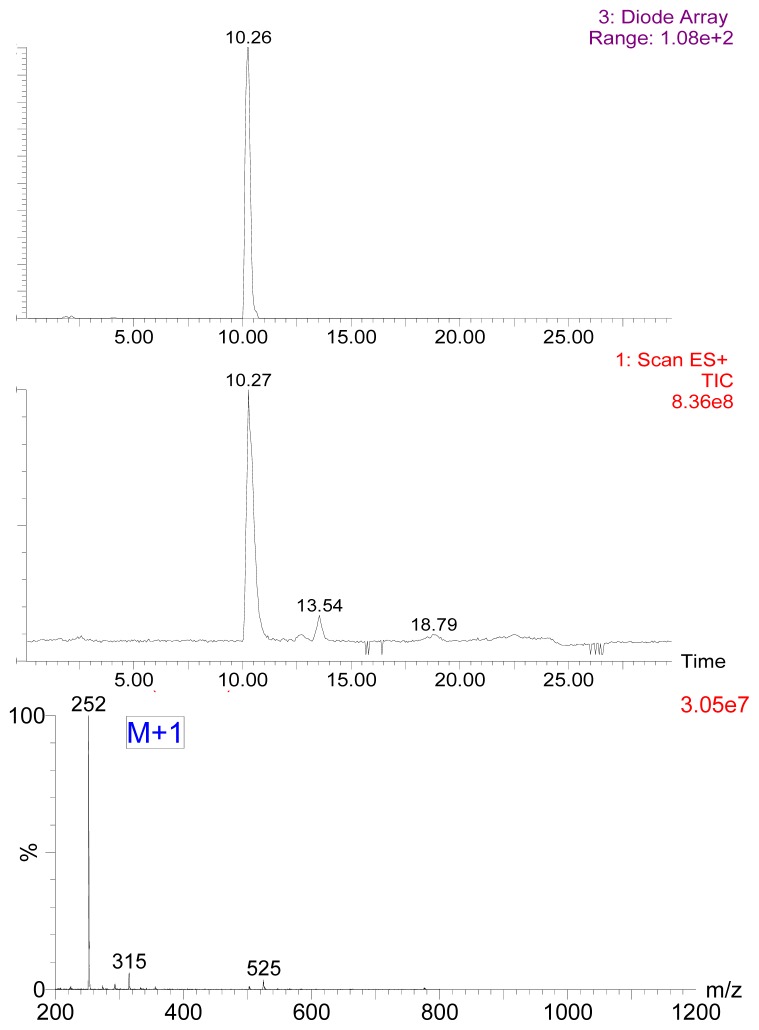
Identification of 7-ANZP by HPLC-ESI-MS.

In order to confirm whether 7-aminonitrazepam (7-ANZP) had been obtained, the product obtaining by reducing process of nitrazepam and subsequent preliminary purification was separated by chromatographic solution hexane/acetic acetate (1:1, *v/v*) on silica TLC plate. Two main bands were obtained, whose *R*_f_ values were 0.72 and 0.35, respectively. The band with *R*_f_ = 0.72 was mainly nitrazepam, while another band with *R*_f_ = 0.35 was 7-ANZP. HPLC-ESI-MS was also utilized to confirm the structure of 7-ANZP (Figure 1). A prominent ion peak (M + 1, *m/z* 252) of 7-ANZP molecule could be clearly observed by HPLC-MS analyzing (Figure 1).

**Figure 2:**
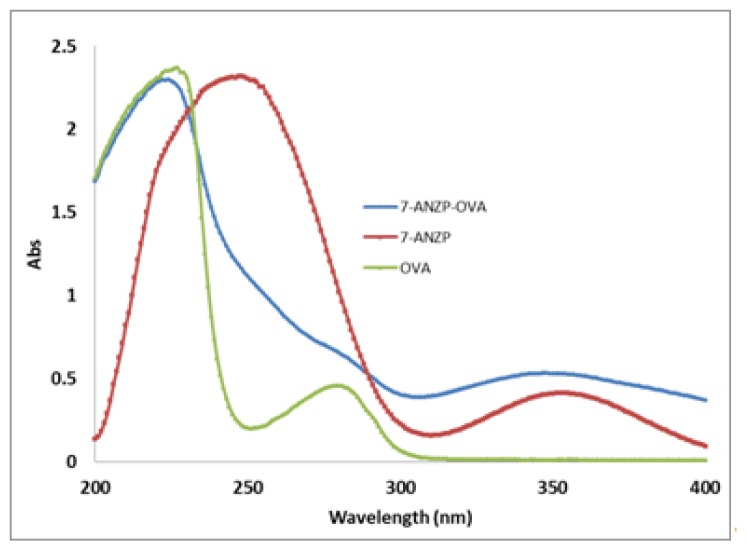
Identification of 7-ANZP coating antigen by UV-vis spectrum.

On the UV-vis spectrum of ANZP-OVA conjugate, an absorbance peak was observed at 355 nm, which is one of the characteristic peaks of 7-ANZP. An absorbance maximum was also found at 245 nm along with a smaller peak at 280 nm, which integrated the characteristic peak (245 nm) of 7-ANZP and the characteristic peak (280 nm) of OVA (Figure 2). These changes on UV-vis spectra showed that the conjugate, ANZP-OVA, could be used for further ELISA development.

### 2.2. Characterization of GNPs and GNPs-IgG-HRP Bioconjugate

**Figure 3:**
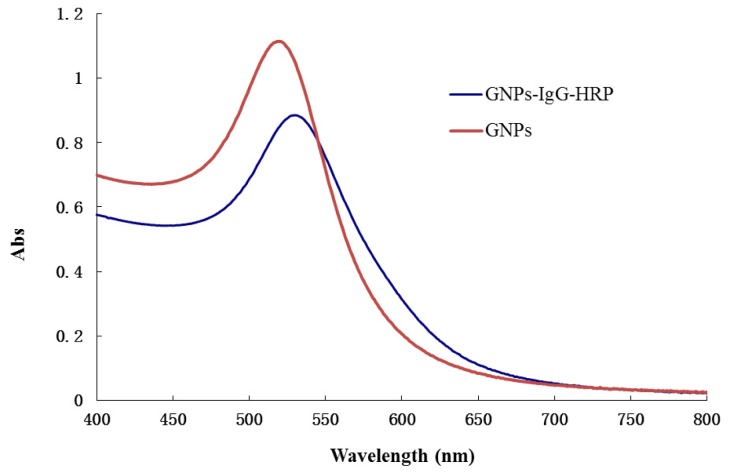
UV-vis spectra of GNPs and GNPs-IgG-HRP.

A novel enzyme tracer was prepared by conjugating IgG-HRP with GNPs. The initial maximum absorbance peak of the GNPs at 520 nm was shifted to 529 nm after conjugation with IgG-HRP molecules (Figure 3), which can be expected based on the local surface plasmon resonance phenomenon [13]. This size expansion indicated the successful conjugation of the GNPs and IgG-HRP. It was important to note that the GNPs -IgG-HRP conjugate was also mono-disperse (data not shown), which will contribute to the reproducibility of the GNPs-based ELISA method.

### 2.3. Nano-Enhanced ELISA

**Figure 4:**
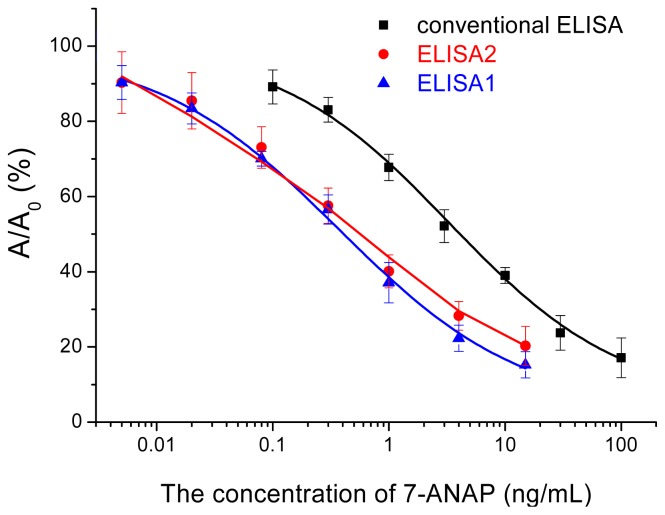
Calibration curve of ELSIA methods for 7-ANZP. Nano-enhanced ELISA1 and ELISA2 were carried out with buffer and simply pretreated urine as matrix, respectively.

For application of the GNPs-IgG-HRP conjugate in ELISA format, the concentrations of coating antigen, anti-7-ANZP antibody and the GNPs-IgG-HRP conjugate firstly were selected by the checkerboard method. Blocking buffer containing proteins or polymers (ovalbumin, gelatin from pork skin, polyvinyl pyrrolidone (PVP, average mw 40 kDa, PEG-10000) were also compared to obtain highest signal to noise ratio. Blocking buffer containing gelatin (0.1%) was chosen (data not shown). Under optimized concentrations of coating antigen, anti-7-ANZP antibody and the GNPs -IgG-HRP conjugate, the sensitivity (defined as the concentration when *A*/*A*_0_% was 90%) could reach 5.6 pg/mL, which was nearly 20 times more sensitive than that of the traditional ELISA for 7-ANZP (Figure 4). The linear range of this nano-ELISA was 0.01~4 ng/mL (the linear equation was *y* = −24.338 × lg*x* + 39.608, *R*^2^ > 99%). These results demonstrated multiple IgG-HRP molecules loading onto the surface of GNPs had endowed the conjugate producing signal amplification effect. The signal amplification effect in this method was close to that of another GNPs-based ELISA for heavy ion [19]. Note that the performance of this nano-enhanced ELISA can be improved further through more elaborate design, for example, optimizing the size of GNPs, adopting other kinds of bigger nanoparticles, or directly conjugating IgG and HRP to improve the loading of HRP molecules per GNP.

Intra-assay and inter-assay precision of the ELISA method were studied. Intra-assay precision was investigated by evaluating the variation of determination of 7-ANZP from well to well (*n* = 5) in the same microplate and inter-assay precision was calculated from measurements in several microplates (*n* = 3). The maximum coefficient of variation (*CV*) of intra-assay was 4.7%, while that of inter-assay was 4.6% (Table 1).

### 2.4. Validation of the Nano-Enhanced ELISA

To investigate whether this nano-enhanced ELISA method could be used in real samples, the matrix effect of human urine was first tested. Considering many metabolites of benzodiazepines are excreted in urine as glucuronide conjugates, we chose β-glucuronidase to gently hydrolyze the possible glucuronide conjugates of 7-ANZP [20]. This enzymatic hydrolysis can potentially be replaced by faster acid hydrolysis.

Pretreated control human urine samples were utilized to prepare 7-ANZP standard solutions for this method. The differences of average values measured were less than 15% in the linear range from 0.01 to 4.0 ng/mL when comparing ELISA1 with ELISA2. ELISA1 was carried out with PBS buffer while ELISA2 used standards using negative urine sample matrix, which was enzymatically hydrolyzed and then diluted 5 fold by PBS buffer. The standard curve obtained using this diluted enzymatically-treated negative control as matrix was almost identical to the curve obtained using assay buffer alone (Figure 4). These results showed the matrix effect may be eliminated almost completely after enzymatic hydrolysis and appropriate dilution.

To evaluate the accuracy and precision of this novel ELISA method, the spiked human urine sample were tested by the established sample pretreatment protocol. After measuring 10 blank samples, the LOD for 7-ANZP in human urine, which is defined as the mean value of these samples plus three standard deviations of their measured signal values, was found to be 0.18 ng/mL. When detecting the urine samples spiked with 0.5, 1.5 and 4 ng/mL 7-ANZP, recoveries ranging from 83.4 to 95.5% were obtained and the relative standard deviations (RSD) were less than 11% (Table 2). These results demonstrated this nano-enhanced ELISA was reliable for 7-ANZP detection in human urine.

In order to verify the applicability of this method in a real situation, the nano-enhanced ELISA method was used in analyzing 3 positive urine samples from patients. The 7-ANZP values of positive samples were found to be 0.58, 0.89 and 2.12 ng/mL, respectively. These urine samples were further confirmed by LC-MS-MS method and the values obtained were 0.51, 0.78 and 1.95 ng/mL, respectively. It seemed the values obtained by this ELISA were all higher than that confirmed by LC-MS-MS method, but the differences were all less than 14%. Considering all the values were at ppb level and the samples were pretreated by a simple way, this difference should be acceptable.

## 3. Experimental Section

### 3.1. Materials

Polycolonal rabbit antibody against 7-aminonitrazepam (7-ANZP) was produced in our lab. 7-aminonitrazepam (7-ANZP) was purchased from National Institutes for food and drug Control (NIFDC, Beijing, China). Ovalbumin (OVA), 3,3′,5,5′-tetramethylbenzidine (TMB), chloroauric acid (HAuCl_4_) and trisodium citrate were purchased from Sigma-Aldrich (St. Louis, MO, USA). The peroxidase-conjugated goat anti-mouse IgG (IgG-HRP) was purchased from Kangchen Biotechnology Company (Shanghai, China). Microtiter plates were obtained from Costar Group, Inc., (Bethesda, MD, USA). Other chemical regents were all analytical grade and from Shanghai Chemical Reagents Company (Shanghai, China). All buffers used in the experiments were prepared using Milli-Q water from Milli-Q system (Millipore, Bedford, MA, USA).

### 3.2. Solutions and Buffers

Coating buffer, 50 mmol/L carbonate buffer (pH 9.5); dilution buffer (phosphate buffered saline (PBS)), 10 mmol/L sodium phosphate buffer (pH 7.4) containing 140 mmol/L NaCl; washing buffer, PBS containing 0.05% (*v/v*) Tween 20 (PBST); TMB solution, 50 mmol/L sodium citrate buffer (pH 5.0) containing 0.01% (*w/v*) TMB and 0.005% (*v/v*) H_2_O_2_.

### 3.3. Preparation of Hapten and Hapten-Protein Conjugate

The hapten of 7-ANZP and protein conjugate were prepared according to the reference in which 7-aminochlonazepam, an analogue of 7-ANZP, was prepared [4]. Briefly, 1.68 g iron powder and 200 mg ammonium chloride were suspended in 5 mL water and heated for 15 min, after which 2.8 g nitrazepam in 100 mL methanol was added to the mixture. After refluxing for 5 h, iron powder was removed by filtration and the methanol was evaporated in vacuum. The precipitate was collected and recrystallized with methanol to give 2.24 g (2.77 g) of fawn crystalloid.

The 7-ANZP-protein conjugate was prepared according to the diazotization method. 10 mg 7-aminonitrazepam was dissolved in warm 0.05 M sulfuric acid (4 mL, 70 °C). The solution was then cooled in an ice bath, 1 mL of freshly prepared sodium nitrate (19 mg/mL) was added dropwise within 3 min and stirred for 30 min. An OVA solution (100 mg in 4 mL of 0.5 M borate, pH 9.4) was cooled down to 0 °C, and the diazotized 7-ANZP solution was added to the cooled OVA solution within 15 min. The pH of the solution was maintained between 9.0 and 9.5 by adding sodium hydroxide (1 M). After 4 h, the reaction mixture was stood at room temperature, exhaustively dialyzed against 10 mM phosphate buffer (pH 7.2, 9 × 2 L) at 4 °C and stored at −20 °C prior to use.

### 3.4. Synthesis and Characterization of Gold Nanoparticles and GNPs-IgG-HRP Conjugate

Gold nanoparticles (20 nm) were prepared by reducing HAuCl4 with trisodium citrate. Flasks and magnetic stir bars used were cleaned sufficiently with aqua regia. 100 mL of 0.1% HAuCl_4_ solution was boiled and then vigorously stirred in round-bottom flask. Then 2 mL of 1% trisodium citrate solution was added quickly to the solution. After a color change from pale yellow to dark red, which indicated the formation of GNPs, the solution was maintained for another 10 min at boiling temperature and then stirred to cool down.

The GNPs-IgG-HRP conjugate was prepared as follow. Exactly 1 mL of GNP (20 nm) solution was mixed with 1, 2 or 3 μL of K_2_CO_3_ (200 mM), and then 100 μL of IgG-HRP solution (100 μg/mL) was added to 1 mL of each of the treated GNP solution. Each mixture was stirred for 30 min and then centrifuged at 15,000 × *g* for 15 min at 4 °C to remove the excess of antibody. The clear supernatant was carefully removed, and the precipitated GNPs conjugates were resuspended in 1 mL of PBS buffer (0.01 M, pH 7.4) and stored at 4 °C. The conjugates prepared through the addition of different amounts of K_2_CO_3_ were evaluated by adding 100 uL of NaCl (10%) and observing their color change. After 24 h at 4 °C, the conjugates prepared with 1 or 2 μL of K_2_CO_3_ appeared blue to some extent, while the conjugates prepared with 3 uL of K_2_CO_3_ still appeared red. Finally, this conjugate, prepared by adding 3 μL of K_2_CO_3_, was chosen for further use. The UV-vis spectroscopy of the conjugate was analyzed to evaluate the size change.

### 3.5. Nano-Enhanced ELISA

The procedure of the nano-enhanced ELISA was almost the same as that of a traditional indirect ELISA method except that the IgG-HRP was replaced with the GNPs-IgG-HRP conjugate. The general procedure developed in the assay was as follows. 100 μL of 7-ANZP-OVA (15 μg/mL) was added to each well of a 96-well microplate and immobilized at 37 °C for 2 h. Coating antigen was decanted and microplates were washed by PBST. The microplates were then blocked by incubating with 200 μL of blocking buffer (1% BSA in PBST) for 2 h at room temperature. After decanting the blocking buffer and washing the microplates, 50 μL of 7-ANZP standard solutions, or test samples, and 50 μL of anti-7-ANZP antibody solution in PBST buffer containing 0.1% gelatin were added to the wells. The microplates were then incubated at 37 °C for 30 min. The reaction mixtures in wells were then decanted and the wells were washed, then 100 μL of diluted GNPs-IgG-HRP conjugate was added to the each well. After incubation for 30 min, the wells were washed again. HRP substrate containing TMB and H_2_O_2_ solutions was added and color was developed for 15 min. Then the reactions were stopped by addition of 2 M sulfuric acid (100 μL) to each well. The optical density was read at 450 nm with a microplate reader within 5 min after stopping the reaction.

### 2.6. Sample Pretreatment and Validation of the Nano-ELISA

Blank urine samples were obtained from healthy individuals as negative samples. Urine samples, collected from 3 patients at the Fourth Hospital in Wuxi who were known to be receiving oral doses of clonazepams (CZP), were analyzed as positive samples. The experiment was approved by the local ethics committee of the hospital.

The urine samples were filtered through Whatman no. 1 filter paper, and stored frozen (−20 °C) in aliquots of 10 mL until required for analysis. Aliquot of 1.0 mL urine sample was centrifuged at 5000× *g* for 10 min at room temperature and to 0.5 mL of the supernatant 0.5 mL of β-glucuronidase (6000 units) was added and incubated for 2.5 h to achieve hydrolysis of the conjugates. Then the hydrolysate was diluted further with phosphate buffer (0.1 M, pH 7.4) for analysis. For validation of the assay, 7-ANZP standard solution (0.1 mg/mL) was added to the negative urine sample in order to obtain 7-ANZP spiked samples. After standing for 30 min, the spiked samples were centrifuged, hydrolyzed and diluted as the above method.

## 4. Conclusions

A nano-enhanced ELISA method, based on GNPs-IgG-HRP conjugates as signal probes, was developed for highly sensitive detection of 7-ANZP in human urine. The preparation of GNPs-IgG-HRP conjugate is facile and the product stable. The procedure of the nano-enhanced ELISA was as simple as a traditional ELISA, but 20 times more sensitive. These results suggest that utilizing GNPs-IgG-HRP conjugate as signal probe to replace IgG-HRP in traditional ELISA is an effective strategy in developing sensitive detection of 7-ANZP in urine samples. This method can also be extended to development of ultrasensitive detection of other small molecules in urine.

## Figures and Tables

**Table 1:** Intra-assay and inter-assay precision of 7-ANZP analyses by the nano-enhanced ELISA.

7-ANZP(ng/mL)	CV (%)	

	Intra-assay	Inter-assay
0.005	4.8	5.2
0.02	4.2	4.7
0.08	1.9	4.8
0.3	3.8	5.5
1	4.3	3.6
4	3.5	4.4
15	5.5	3.9

**Table 2:** Recovery of 7-ANZP in urine detected by the nano-ELISA (*n* = 4).

Spiked concentration (ng/mL)	Results measured (ng/mL)	Recovery (%)	RSD (%)
0.5	0.417 ± 0.039	83.4	7.8
1.5	1.352 ± 0.141	90.1	9.3
4.0	3.823 ± 0.405	95.5	10.1

## References

[1] Laurie D., Mason A.J., Piggott N.H., Rowell F.J., Seviour J., Strachan D., Tyson J.D. (1996). Enzyme linked immunosorbent assay for detecting benzodiazepines in urine. Analyst.

[2] Kuang H., Li Q., Shen C., Xu J., Yuan Y., Xu C., Wang W (2009). A highly sensitive method for the determination of 7-aminonitrazepam, a metabolite of nitrazepam, in human urine using high-performance electrospray liquid chromatography tandem mass spectrometry. Biomed. Chromatogr.

[3] Moriya F., Hashimoto Y (2003). Tissue distribution of nitrazepam and 7-aminonitrazepam in a case of nitrazepam intoxication. Forensic Sci. Int.

[4] Elian A.A. (2003). ELISA detection of clonazepam and 7-aminoclonazepam in whole blood and urine. Forensic Sci. Int.

[5] Zhu Y., Tan J.Y., Sun Y.Q. (2003). Determination of 7-aminonitrazepam, metabolite of nitrazepam, in urine by tert-butyldimethylsilyl derivatization-gas chromatography-mass spectrometry. Chin. J. Anal. Chem.

[6] Smink B.E., Brandsma J.E., Dijkhuizen A., Lusthof K.J., de Gier J.J., Egberts A.C.G., Uges D.R.A. (2004). Quantitative analysis of 33 benzodiazepines, metabolites and benzodiazepine-like substances in whole blood by liquid chromatography-(tandem) mass spectrometry. J. Chromatogr. B-Anal. Technol. Biomed. Life Sci.

[7] Miller E.I., Wylie F.M., Oliver J.S. (2006). Detection of benzodiazepines in hair using ELISA and LC-ESI-MS-MS. J. Anal. Toxicol.

[8] Nakamura M (2011). Analyses of benzodiazepines and their metabolites in various biological matrices by LC-MS(/MS). Biomed. Chromatogr.

[9] Yue N., Wu L., Li L., Xu C (2009). Multi-residue detection of benzodiazepines by ELISA based on class selective antibodies. Food Agric. Immunol.

[10] Chen W., Peng C., Jin Z., Qiao R., Wang W., Zhu S., Wang L., Jing Q., Xu C (2009). Ultrasensitive immunoassay of 7-aminoclonazepam in human urine based on CdTe nanoparticle bioconjugations by fabricated microfluidic chip. Biosens. Bioelectron.

[11] Jiang J., Wang Z., Zhang H., Zhang X., Liu X., Wang S (2011). Monoclonal antibody-based ELISA and colloidal gold immunoassay for detecting 19-nortestosterone residue in animal tissues. J. Agric. Food Chem.

[12] Liu N., Han Z., Lu L., Wang L., Ni G., Zhao Z., Wu A., Zheng X (2013). Development of a new rabbit monoclonal antibody and its based competitive indirect enzyme-linked immunosorbent assay for rapid detection of sulfonamides. J. Sci. Food Agric.

[13] Yeh C.-H., Hung C.-Y., Chang T., Lin H.-P., Lin Y.-C. (2009). An immunoassay using antibody-gold nanoparticle conjugate, silver enhancement and flatbed scanner. Microfluid. Nanofluid.

[14] Zhang Y., Pan T., Fang G., Ma D., Wang S (2009). Development of a solid-phase extraction-enzyme-linked immunosorbent assay for the determination of 17beta-19-nortestosterone levels in antifatigue functional foods. J. Food Sci.

[15] Peng C.-F., Chen Y.-W., Chen W., Xu C.-L., Kim J.-M., Jin Z.-Y. (2008). Development of a sensitive heterologous ELISA method for analysis of acetylgestagen residues in animal fat. Food Chem.

[16] Ambrosi A., Airò F., Merkoçi A (2009). Enhanced gold nanoparticle based ELISA for a breast cancer biomarker. Anal. Chem.

[17] Cui G., Chu H., Hu Y., Xu C (2010). Ultrasensitive signal amplified immunoassay of medroxyprogesterone acetate (MPA) using the atomic absorption of silver deposited on the surface of gold nanoparticles. Food Agric. Immunol.

[18] Mei Z., Chu H., Chen W., Xue F., Liu J., Xu H., Zhang R., Zheng L (2013). Ultrasensitive one-step rapid visual detection of bisphenol A in water samples by label-free aptasensor. Biosens. Bioelectron.

[19] Zhou Y., Tian X.L., Li Y.S., Zhang Y.Y., Yang L., Zhang J.H., Wang X.R., Lu S.Y., Ren H.L., Liu Z.S. (2011). A versatile and highly sensitive probe for Hg(II), Pb(II) and Cd(II) detection individually and totally in water samples. Biosens. Bioelectron.

[20] Borrey D., Meyer E., Lambert W., van Peteghem C., de Leenheer A.P. (2001). Simultaneous determination of fifteen low-dosed benzodiazepines in human urine by solid-phase extraction and gas chromatography—mass spectrometry. J. Chromatogr. B.

